# Case report: An endoscopic approach to the middle ear following significant penetrating trauma

**DOI:** 10.1016/j.ijscr.2025.111883

**Published:** 2025-09-02

**Authors:** Alison McHugh, Khalid Majeed, Orla Young, Ivan Keogh

**Affiliations:** a Department of Otolaryngology, University Hospital Galway, Ireland; b Discipline of Otorhinolaryngology, University of Galway, Ireland; c Royal College of Surgeons, Ireland

**Keywords:** Penetrating, ‘Middle ear’, Trauma, Endoscopy, Stapes, Endolymph

## Abstract

**Introduction and importance:**

While blunt trauma to the head is a well-recognized cause of middle ear injuries, penetrating traumas are far less common. Due to the close anatomical relations, the potential consequences of such injuries can be catastrophic.

**Case presentation:**

A man presented following a penetrating injury to the external auditory canal. He sustained unilateral sudden hearing loss, tinnitus, imbalance and facial nerve palsy.

**Discussion:**

Radiological imaging showed a pneumolabyrinth and suggested middle ear foreign body. Endoscopic middle ear exploration was undertaken, demonstrating adhesions and organic debris, a dehiscent and oedematous facial nerve, downward fracture of the stapes footplate and a perforation in the oval window. Endoscopic debridement with facial nerve decompression and fat plug graft of the oval window allowed full recovery of facial nerve function and improvement of vestibular symptoms.

**Conclusion:**

Endoscopic ear surgery can be a valuable tool for exploring and repairing penetrating middle ear injuries.

## Highlights

• Penetrating middle ear trauma is rare and can result in a myriad of catastrophic injuries due to the complex anatomy and proximity to critical structures including the otic capsule, facial nerve and otic capsule
• Management approach should include urgent ENT referral, audiometry and cross sectional imaging to evaluate the extent of injury. Treatment strategies include an intact tympanic membrane, normal vestibular function and hearing and complete facial nerve function.
• Endoscopic exploration of the middle ear in penetrating trauma is rarely described. An endoscopic approach to the middle ear can offer advantageous views when exploring middle ear injuries with minimal additional morbidity. Post-auricular wound and scarring are avoided with lower pain anticipated and shorter post-operative recovery.
• Endoscopic exploration allowed for identification of ossicular disarticulation and fracture, facial nerve dehiscence and perilymphatic fistula. Endoscopic facial nerve decompression restored facial nerve function and endoscopic repair of the perilymphatic fistula with fat plug grafting restored vestibular function. Endoscopic ear surgery proved effective and advantageous in this case, highlighting its value in managing complex ear trauma.

## 1 Introduction

Penetrating middle ear injuries are rare but potentially catastrophic due to the intricate anatomy of the middle ear. Sequelae may include hearing loss, imbalance, facial palsy, cerebrospinal fluid leakage and perilymph fistula, warranting an early expert otolaryngology opinion for the best chance of recovery [1]. An endoscopic approach to the ear can allow for detailed visualisation with minimal additional morbidity in cases of penetrating middle ear trauma. This case report has been reported in line with the SCARE checklist [2].

## 2 Case report

A 32 year old male presented acutely to a tertiary referral centre. While trying to maneuver between his parked van and a bush, a branch penetrated his left ear canal, causing immediate disabling dizziness. He had sudden onset complete ipsilateral facial weakness and hearing loss. Feeling too dizzy to stand, he was brought to hospital by ambulance. He had a background history of type 1 diabetes mellitus and worked in the building industry.

On arrival to the hospital, his facial nerve palsy had improved. Otoscopy showed a clot posteriorly in the left external auditory canal without any obvious foreign body. He had a moderate to severe conductive hearing loss on audiogram. With ongoing nausea, vomiting and ataxia, he was admitted for fluid resuscitation, antiemetics and glycaemic control. He was commenced on oral steroids.

**Fig. 1:**
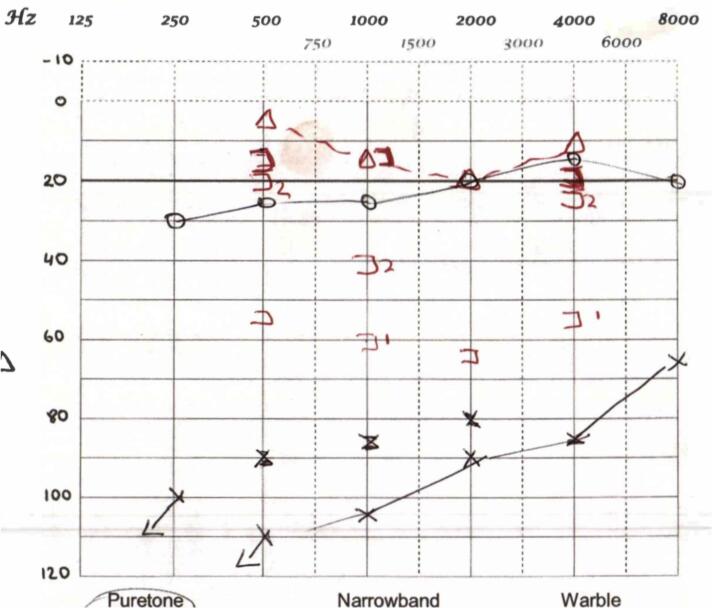
Audiogram showing profound left sensorineural hearing loss, with inconsistent responses due to significant tinnitus.

Over the following week, he had very little response to treatment with progressive hearing loss, imbalance and worsening facial weakness. His facial nerve palsy deteriorated to House Brackmann VI (Table 1). He was ataxic and unable to return to work. An audiogram confirmed progressive hearing loss with inconsistent responses incurred by profound tinnitus (Fig. 1).

**Table 1:** House Brackmann Scale of Facial Nerve Palsy [*modified from House et al, 1985* [11]]*.*

Grade	Description	Characteristics
1	Normal	Normal facial function
2	Mild dysfunction	Slight weakness in inspection; symmetric at rest
3	Moderate dysfunction	Obvious weakness ± asymmetry at rest but not disfiguring. Can complete eye closure with effort
4	Moderate/severe dysfunction	Obvious weakness or disfiguring asymmetry. Unable to complete eye closure with effort
5	Severe dysfunction	Barely perceptible movement. Disfiguring asymmetry at rest
6	Total paralysis	No movement

**Fig. 2:**
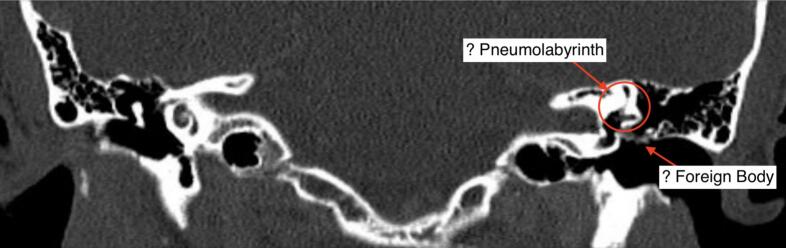
Coronal CT Temporal Bones on bony windows showing airs within the left labyrinthine system.

Magnetic resonance imaging (MRI) of the brain and internal auditory meatus (IAM) and computed tomography (CT) of the head and temporal bones were performed. On these, air could be appreciated within the labyrinthine system (Fig. 2).

At this stage he was transferred to the care of the otology service. The patient was taken to theatre for otoendoscopic exploration of the left middle ear. Endoscopic exploration was undertaken using a 3 mm 14 cm 0 degree Hopkins rod and specialised set of endoscopic middle ear instruments.

**Fig. 3:**
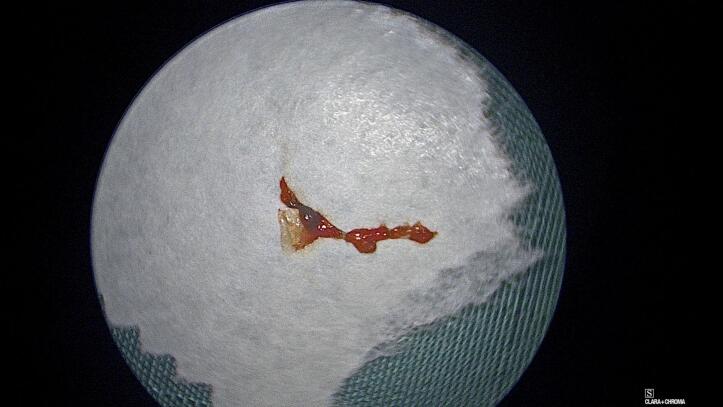
Clot and organic debris cleaned from the middle ear.

**Fig. 4:**
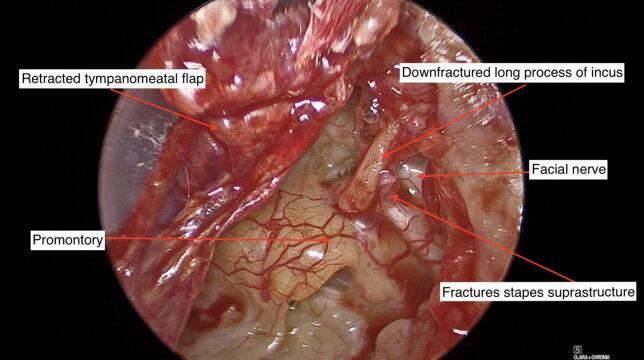
Otoendoscopic view of the left middle ear showing a medialised & down-fractured long process of incus and fractured stapes suprastructure.

On elevation of a tympanomeatal flap, the middle ear was noted to be full of scar tissue and adhesions. A clot with organic debris was identified just deep to the tympanic membrane (Fig. 3). This was removed and the area cleaned. The adhesions were divided. A flattened, hypermobile incus became obvious. The stapes suprastructure was fractured downward (Fig. 4).

**Fig. 5:**
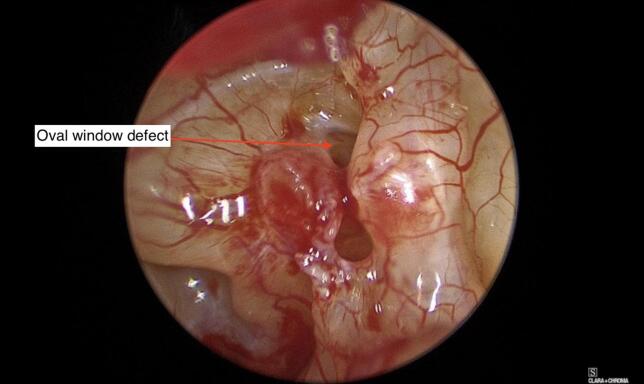
Oval window defect with perilymph fistula.

Further exploration revealed a large perforation in the oval window (Fig. 5).

**Fig. 6:**
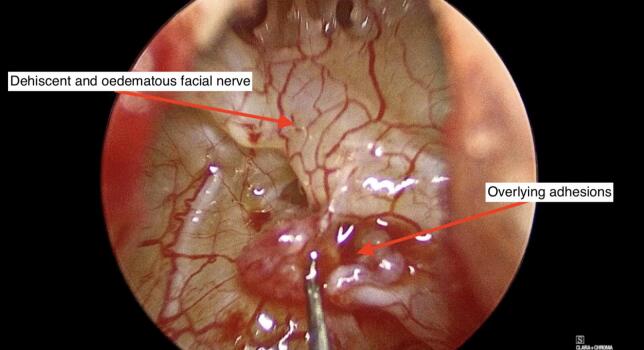
Dehiscent facial nerve in the middle ear with overlying adhesions.

The redundant incus was removed. Further adhesions found along the facial nerve which were divided. Although oedematous and dehiscent throughout the tympanic segment, the facial nerve was found to be intact (Fig. 6). However, it was not stimulating upon completion of the procedure.

**Fig. 7:**
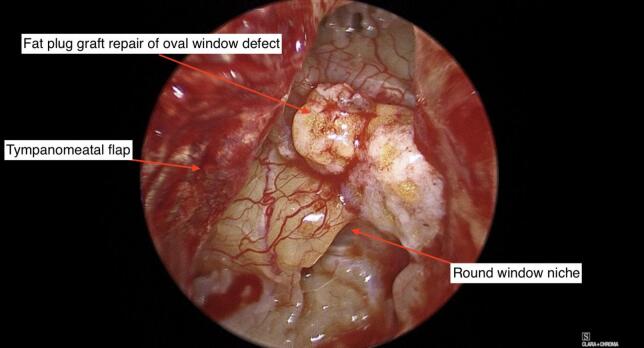
Repair of oval window defect with fat plug graft.

The defect in the oval window was repaired with a fat graft from the ear lobule (Fig. 7). A standard closure was done by filling the middle ear was filled with gelfoam and replacing the tympanomeatal flap was with Biodesign**®** underlay.

The patient made an uncomplicated post-operative recovery. By two weeks, his balance had improved. His facial nerve function slowly improved, returning to House Brackmann Grade IV four weeks post-operatively and by eighteen months to House Brackmann Grade I. Unfortunately, he was left with profound sensorineural hearing loss.

## 3 Discussion

Penetrating injuries to the middle ear are uncommon but bear significant potential morbidity. A vast array of penetrating objects have been described, including twigs, iatrogenic instruments, hair combs tails and pins and knitting needles [[3], [4], [5]]. Due to the complex anatomy and important relations of the middle ear, injuries may occur to a number of important structures such as the otic capsule, facial nerve, tegmen, jugular bulb and carotid artery. Consequently, these injuries can be catastrophic. The goals of treatment include restoring an intact tympanic membrane, improving hearing and facial nerve function and vestibular rehabilitation. Those presenting with marked hearing loss, facial nerve dysfunction or vestibular dysfunction warrant urgent ENT evaluation [1]. Comprehensive clinical and neuro-otological examination should be undertaken including pure tone audiometry. A fine cut CT scan of the temporal bone should be performed. If indicated, an MRI IAM allows for closer scrutiny of the facial nerve. Pneumolabyrinth is discrete on imaging and must be closely examined for if suspected [6].

Management approaches are tailored to the injuries sustained. Topical antibiotics should be administered with consideration to systemic antibiotics where significant contamination is suspected [7]. In cases with facial nerve palsy or suspected labyrinthine injury, if no contraindication, systemic steroids should be given. In cases with protracted nausea or vomiting anti-emetics and possibly intravenous fluid replacement may be warranted. Vestibular sedatives may be of benefit and have a low side-effect profile.

Surgical exploration is indicated where there is an retained foreign body or contamination that requires removal, ossicular disruption needing repair, facial nerve injury warranting exploration, suspected perilymph fistula, otic capsule injury or CSF leak needing repair [1,7]. A middle ear exploration can be undertaken endoscopically, as in our case. Endoscopic explorations of the middle ear in such traumatic cases have rarely been reported. Endoscopic ear surgery allows a greater field of vision with angled cameras visualising areas that may out of reach of a microscope [8,9]. Further benefits to the patient include avoidance of a post-auricular scar and potentially shorter operative time [9]. Where additional access is required, for example for nerve repair or to safely remove a foreign body, a post-auricular or combined approach may be needed. All possible injuries should be anticipated pre-operatively and prepared for.

Outcomes are dependent on the injury sustained. Conductive components of hearing offer the possibility of ossicular chain reconstruction while sensorineural losses tend to be permanent [1,10]. Outcomes for facial nerve injuries are variable. In this case, the patient experienced an initial sudden complete nerve palsy at the time of trauma. As the nerve was intact, this transiently improved but later again deteriorated, likely due to localized irritation and oedema. Once surgically cleared of debris and decompressed, this then recovered. Decompression offers better outcomes. Electroneurography is useful in predicting recovery [4]. Persistent facial nerve palsies are managed dependant on both the injury and patient factors with options including observation, nerve graft or repair, and static or dynamic reconstruction. For traumatic perilymphatic fistulae, balance often recovers after a successful repair [1,5,6]. However, this is typically accompanied by a progressive sensorineural hearing loss to a profound nerve hearing loss, as seen in our case [1].

In summary, we present a challenging case of significant middle ear trauma secondary to a penetrating injury. Injuries sustained were life changing. Endoscopic exploration allowed detailed examination of the middle ear and clearly detailed injuries sustained. Endoscopic debridement, facial nerve decompression and repair of the oval window perforation have offered this patient the best outcomes. There are very few reports of endoscopic exploration of the middle ear post penetrative trauma. Endoscopic exploration is a viable and valuable option for the ear surgeon.

## Consent

Written informed consent was obtained from the patient for publication and any accompanying images. A copy of the written consent is available for review by the Editor-in-Chief of this journal on request.

## Ethical approval

This study was exempt from ethical approval at University Hospital Galway due to the nature of it being a case report with written consent from the patient.

## Funding

N/A.

## Author contribution

All authors have contributed to the paper's data collection and analysis, drafting and revision of the paper.

## Guarantor

Alison McHugh.

## Research registration number

N/A.

## Conflict of interest statement

The authors disclose no conflicts of interest.
